# Identification of Functional Domains in the Cohesin Loader Subunit Scc4 by a Random Insertion/Dominant Negative Screen

**DOI:** 10.1534/g3.116.031674

**Published:** 2016-06-07

**Authors:** Michal Shwartz, Avi Matityahu, Itay Onn

**Affiliations:** Faculty of Medicine in the Galilee, Bar-Ilan University, Safed, 1311502, Israel

**Keywords:** *SCC4*, *SCC2*, loader, cohesin, SMC

## Abstract

Cohesin is a multi-subunit complex that plays an essential role in genome stability. Initial association of cohesin with chromosomes requires the loader—a heterodimer composed of Scc4 and Scc2. However, very little is known about the loader’s mechanism of action. In this study, we performed a genetic screen to identify functional domains in the Scc4 subunit of the loader. We isolated scc4 mutant alleles that, when overexpressed, have a dominant negative effect on cell viability. We defined a small region in the N terminus of Scc4 that is dominant negative when overexpressed, and on which Scc2/Scc4 activity depends. When the mutant alleles are expressed as a single copy, they are recessive and do not support cell viability, cohesion, cohesin loading or Scc4 chromatin binding. In addition, we show that the mutants investigated reduce, but do not eliminate, the interaction of Scc4 with either Scc2 or cohesin. However, we show that Scc4 cannot bind cohesin in the absence of Scc2. Our results provide new insight into the roles of Scc4 in cohesin loading, and contribute to deciphering the loading mechanism.

The initial association of cohesin with chromatin depends on the loading complex encoded by the *SCC4* and *SCC2*/*NIPBL* genes (Ciosk *et al.* 2000; Watrin *et al.* 2006). Cohesin mediates long-range chromatin interactions, and is essential for maintaining genome integrity (Onn *et al.* 2008; Jeppsson *et al.* 2014; Marston 2014). During the cell cycle, cohesin ensures accurate segregation of the sister chromatids by tethering them from the time of their replication until their separation during mitosis. In addition, the cohesin loader is associated with nonmitotic cellular and developmental processes, as well as clinical disorders. Cohesin is important for DNA repair, and for the regulation of gene expression (Onn *et al.* 2008; Jeppsson *et al.* 2014; Marston 2014). Scc2 promotes gene expression, and mutations in the gene were identified as the main cause for the developmental disorder Cornelia de Lange syndrome (CdLS) (Krantz *et al.* 2004). Human *SCC4* is involved in neural development (Smith *et al.* 2014). Despite the unquestioned importance of the loader in cohesin activity, cell functionality and human health, very little is known about the molecular mechanism involved.

The loader proteins Scc2 and Scc4 form a stable dimer that physically interacts with cohesin (Ciosk *et al.* 2000). The crystal structure of Scc4 with the N-terminal fragment of Scc2 reveals that Scc2 is mainly unstructured, and that Scc4 is wrapped around the Scc2 polypeptide (Figure 1A, and Supplemental Material, Figure S2) (Chao *et al.* 2015; Hinshaw *et al.* 2015). Negative staining electron microscopy of the dimer suggests that the loader alternates between extend and compact conformations (Chao *et al.* 2015). Scc2/Scc4 maintains a nucleosome-free region in the DNA, and induces a conformational change in cohesin that enables the chromatin to become entrapped (Ciosk *et al.* 2000; Arumugam *et al.* 2003; Gruber *et al.* 2006; Kurkcuoglu and Bates 2010; Lopez-Serra *et al.* 2014). After loading is completed, ATP is hydrolyzed by the SMC proteins. In turn, cohesin is dissociated from Scc2/Scc4 and is translocated from the loading site (Weitzer *et al.* 2003; Hu *et al.* 2011; Ladurner *et al.* 2014; Murayama and Uhlmann 2015). Some evidence suggests that Scc2 is also colocalized with cohesin on chromosome arms (Kogut *et al.* 2009). Both *SCC4* and *SCC2* are essential genes; however, while Scc2 is required for cohesin loading *in vitro*, Scc4 is expendable (Murayama and Uhlmann 2014). Roles of Scc4 in loading, and in other fundamental matters related to the loading mechanism, are still unknown.

**Figure 1:**
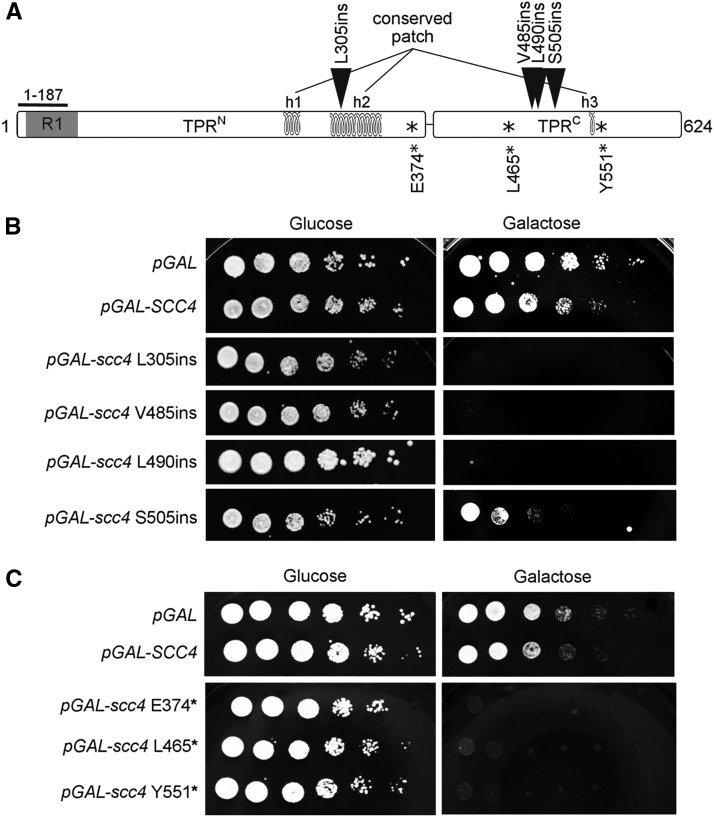
Overexpression of scc4 RIDs inhibits cell growth. (A) Location of Scc4 insertion (triangles) and nonsense (asterisks) mutations identified in this study. The main structural regions are indicated. The 13 TPR repeats are divided into TPR^N^ and TPR^C^, which contain eight and five repeats, respectively. The three helices, h1, h2, and h3, form a tertiary structure conserved patch, which enables essential cohesin loading onto the centromeres. The N-terminus of Scc2 passes through the inner cavity of Scc4 and emerges near the N-terminus of Scc4, where Scc2 residues 112–120 interact with the outer surface of the first Scc4 TPR repeat (R1). (B) Strain YIO002 (*scc4-4*) cells carrying pRS406 (*pGAL URA3*), pIO014 (*pGAL-SCC4 CEN URA3*), pMS011 (*pGAL-scc4-L305ins CEN URA3*), pMS012 (*pGAL-scc4-V485ins CEN URA3*), pMS013 (*pGAL-scc4-L490ins CEN URA3*), or pMS014 (*pGAL-scc4-S505ins CEN URA3*), were grown to saturation in SD-URA medium. Tenfold serial dilutions of each strain were plated on SD-URA plates containing either glucose or galactose and grown at 23°. (C) Strain YIO002 (*scc4-4*) cells carrying pRS406 (*pGAL URA3*), pIO014 (*pGAL-SCC4 URA3*), pMS015 (*pGAL-scc4-E374x CEN URA3*), pMS016 (*pGAL-scc4-L465x CEN URA3*) or pMS017 (*pGAL-scc4-Y551x CEN URA3*) were grown to saturation in SD-URA medium. Tenfold serial dilutions of each strain were plated on SD-URA plates containing either glucose or galactose and grown at 23°.

A model for the molecular mechanism of the loader requires at least three intermolecular interactions: Scc2 and Scc4, Scc2/Scc4 and cohesin, and Scc2/Scc4 and chromatin. The structure of Scc4 in the complex with Scc2 shows that the N-terminal region of Scc2 is important for Scc4 interaction (Chao *et al.* 2015; Hinshaw *et al.* 2015). Multiple contacts between the proteins were reported recently (Chao *et al.* 2015; Hinshaw *et al.* 2015). However, these latter studies did not identify key regions in Scc4 that contribute to the interaction. In yeast, cohesin loading is regulated by a cell cycle-dependent proteolytic cleavage of Scc2. The protein is cleaved after loading in G1/S, and the cleaved product is unable to interact with Scc4 (Woodman *et al.* 2014). Biochemical analysis of Scc2 containing CdLS-associated mutations revealed that some of these mutations alter the interaction of the mutated Scc2 with Scc4. These observations suggest that the intimate interaction between Scc4 and Scc2 is important for proper function of the dimer. Nevertheless, a detailed analysis of the domains in Scc4 is still required to fully understand the properties of the dimer.

The question as to how Scc2/Scc4 interacts with cohesin has yet to be addressed. The interaction may depend on Scc4, Scc2, or Scc2/Scc4 dimerization. Since these proteins are essential, a temporal deletion is required to approach the matter. Recently, we showed that the loader interacts with at least two subunits of cohesin: the regulatory protein Scc3, and one or more subunits of the Smc1–Smc3–Mcd1 trimer (Orgil *et al.* 2015). Studies in *Schizosaccharomyces pombe* also showed multiple interactions between Mis4^Scc2^–Ssl3^Scc4^ and cohesin (Murayama and Uhlmann 2014). These multiple contacts are essential for proper cohesin loading, and Scc4 may be assumed to contribute to one or more of these interactions.

The third predicted activity of the loader is the interaction with chromatin. This interaction may be direct or indirect, through interaction with a chromatin-associated protein. In cells, chromatin structure is an important factor in proper loading of cohesin (Fernius *et al.* 2013; Rudra and Skibbens 2013; Lopez-Serra *et al.* 2014). A helix bundle in Scc4 was shown to promote centromeric cohesion (Hinshaw *et al.* 2015). A mutation that interrupts the interaction had a local effect on cohesin centromeric loading, but not on chromosome arm loading (Hinshaw *et al.* 2015). The contribution of Scc4 to the association of the Scc2/Scc4 dimer, with chromatin in both centromeric and arm regions, needs to be explored.

Scc4 is a 72 kDa protein that contains TPR repeats (Bermudez *et al.* 2012; Chao *et al.* 2015; Hinshaw *et al.* 2015). With the aim of identifying functional regions in Scc4, and testing their importance to the cohesin loading mechanism, we applied a screening strategy called Random Insertion of Dominant negative (RID) (Milutinovich *et al.* 2007; Eng *et al.* 2014; Orgil *et al.* 2015). We constructed a library of scc4 mutant alleles, and used the yeast *Saccharomyces cerevisiae* to isolate functional mutants. We identified five functional regions, and dissected their importance to Scc2/Scc4 function. Our data provide new insight into the mechanism of cohesin loading, and into the specific role of Scc4 in the process.

## Materials and Methods

### Yeast strains and media

Yeast strains and plasmids used in this study are listed in Table S1 and Table S2. Yeast strains were grown in SD-URA or YPD medium, as described, supplemented with 2% glucose (Guthrie and Fink 1991). Medium used for galactose inductions contained SD-URA supplemented with 2% galactose.

### Cell synchronization

Cells were arrested in the G1 phase by the addition of α-factor (1.5 × 10^–8^ M final). To release cells from α-factor-induced G1 arrest, cells were washed twice with YPD medium containing pronase E (0.1 mg/ml; Sigma), and twice with medium without pronase E. Exponentially growing cultures were arrested in G2/M using nocodazole (15 μg/ml final) in the indicated medium.

### RID screen

The construction of the RID library, and the strategy to isolate scc4 mutants is described in Figure S1. In short, a library of mutant plasmids was prepared using the EZ-Tn5 In-Frame Linker Insertion Kit (epicenter, Illumina), on a *CEN3URA3* plasmid bearing *SCC4* under the control of the GAL promoter (pIO014), according to the manufacturer’s instructions. Briefly, an *in vitro* transposon (Tn) insertion reaction randomly inserted a 1100-bp transposon into pIO014. The plasmid library was transformed into bacteria, and a selectable marker on the transposon was used. The Tn was excised by restriction digestion (using a site present at both ends of the Tn), and a new library was made by recircularizing the plasmids, which left an inframe 57-bp insertion at the site of the initial Tn insertion (RID library). The RID library was transformed into haploid strain YIO002 (*scc4-4*). A thermo-sensitive (ts) strain grown at permissive temperature (23°) was used to increase the sensitivity of the screen. Transformants were grown on SD-URA glycerol plates to select for the RID plasmid, but not to induce overexpression of the *SCC4*-RID gene. Transformants were kept to a density of about 150 colonies per plate for ease of screening, and incubated at 23° until colonies formed. We then replica-plated SD-URA plates containing either glucose (noninducing) or galactose (inducing), and incubated at 23°. Colonies that were inviable on galactose were retested to confirm this phenotype. Plasmids were isolated from galactose-sensitive transformants, and the location of the 57 bp insertion was determined by sequencing of the entire gene. To confirm linkage of inviability to candidate RID plasmid, hits were retransformed into strain YIO002 and treated as described above to confirm that the *SCC4*-RID plasmid was responsible for toxicity on galactose medium.

### Immunoprecipitation and Western blotting analysis

Cells were grown to midlog phase, pelleted, washed with dH_2_O, and frozen in liquid nitrogen. Pellets were resuspended in 350 μl IPH50 [50 mM Tris pH 8.0, 50 mM NaCl, 5 mM EDTA, 0.5% NP-40, 5 mM β-mercaptoethanol, protease inhibitor cocktail (Sigma)]. Cells were lysed by adding glass beads (Sigma) to the resuspended pellets followed by four working cycles of 1 min in a bullet blender (Next Advance). The lysates were cleared by two centrifugations of 5 min and 15 min at 1000 × *g* and 14,000 × *g*, respectively, at 4°. Immunoprecipitations were performed at 4° adding the appropriate antibodies for 2 h. The antibodies were collected on protein A/G agarose (Santa Cruz) or on magnetic beads (BIO-RAD) for 1 hr, and washed three times with IPH50 and resuspended in 35 μl Laemmli buffer. Standard procedures for sodium dodecyl sulfate–polyacrylamide gel electrophoresis and Western blotting were followed to transfer proteins from gels to a polyscreen PVDF membrane (Millipore). Membranes were blotted with the primary antibodies. Antibodies were detected using SuperSignal West Pico (Thermo Scientific) and LAS 4000 (GE Healthcare). Antibodies used in this study were: anti-HA (12CA5, Roche), anti-MYC (9E10, Roche), anti-V5 (Invitrogen/Millipore), anti-3Flag (Sigma), and anti-6His (Sigma).

### Site-directed mutagenesis

Site-directed mutagenesis was performed on pMS2 (*SCC4*-3V5, *URA3*) by using Q5 Site-Directed Mutagenesis Kit (New England Biolabs, Inc) following the manufacturer’s instructions. Primers used for the reactions are listed in Table S3.

### Cohesion assay and chromatin immunoprecipitation (ChIP)

Cohesion at *LYS4* was assayed using the LacI-GFP/LacO array. Cells were treated as described in the text and processed to visualize GFP foci by microscopy as described previously (Orgil *et al.* 2015). Each experiment was repeated three times, and at least 300 cells were counted for each time-point in each experimental condition. ChIP was performed as described in Orgil *et al.* (2015). Primers used for qPCR are listed in Table S3.

### Auxin-induced depletion

An auxin-induced degron (AID) system for yeast was previously described (Morawska and Ulrich 2013). Cells were grown to an early midlog phase (OD_600_ = 0.4) in YPD, then split in half. 3-Indoleacetic acid (IAA, Sigma) was diluted in 70% ethanol and added to one-half of the culture to a final concentration of 1 mM. The second culture was not treated with IAA but both halves were incubated in the dark for an additional 1.5 h before being processed for immunoprecipitation as described above.

### Microscopy

Wide-field fluorescence images were obtained using the Zeiss AxioImager M2 fully motorized microscope (100X Plan-Apo, 1.4NA) fitted with an AxioCamHRm CCD High Resolution Camera.

### Data availability

The authors state that all data necessary for confirming the conclusions presented in the article are represented fully within the article.

## Results

### Identification of functional domains in Scc4 by a genetic screen for RID mutants

RID is an efficient strategy, used previously by us and others to isolate mutant alleles of genes encoding cohesin subunits (Milutinovich *et al.* 2007; Eng *et al.* 2014; Orgil *et al.* 2015). A library of *SCC4* RID mutants was prepared using a transposon insertion kit, in which a 57 bp DNA fragment was randomly inserted into a CEN *URA3* plasmid harboring the *SCC4* coding sequence under the control of the GAL promoter (*Materials and Methods* and Figure S1). During preparation of the library, we noticed residual kanamycin-containing plasmids (0.9 CFU/ml). This indicated that cleavage of the transposon was incomplete (Figure S1). The *SCC4* mutant library was transformed into haploid strain YIO002 containing the ts allele *scc4-4*, and cell growth was assayed on both glucose and galactose plates at the permissive temperature (23°). The *scc4-4* ts allele background was used to increase the sensitivity of the screen. We identified seven colonies that exhibited toxicity or lethality when grown on galactose, but that were unaffected when grown on glucose. Plasmids that inhibited growth were isolated and the location of the insertion was determined by sequencing. We identified four plasmids that we collectively called *scc4*-RIDs. These contain the expected 57-bp insertion in the *SCC4* open reading frame after amino acids L305 (*scc4-L305ins*), V485 (*scc4-V485ins*), L490 (*scc4-L490ins*), and S505 (*scc4-S505ins*) (pMS011, pMS012, pMS013, and pMS014, respectively). Unexpectedly, we also isolated three plasmids in which the full transposon was inserted. In these cases, the result of the full-length transposon insertion was a nonsense allele just after the insertion site. The stop codons were located after residues E374 (scc4 374*), L465 (scc4 465*), and Y551 (scc4 551*) (pMS015, pMS016, and pMS017, respectively) (Figure 1A and Figure S2).

To verify the dominant negative effect of the mutants on cell growth, we tested strains containing the GAL-inducible *scc4* mutant alleles by a semiquantitative growth assay. Overexpression of all nonsense alleles, as well as *scc4-L305ins*, *scc4-V485ins*, and *scc4-L490ins* was lethal, while cells overexpressing *scc4-S505ins* showed 1000-fold growth inhibition compared to the control strain (Figure 1, B and C).

### Overexpression of the N-terminal region of Scc4 inhibits cell growth

**Figure 2:**
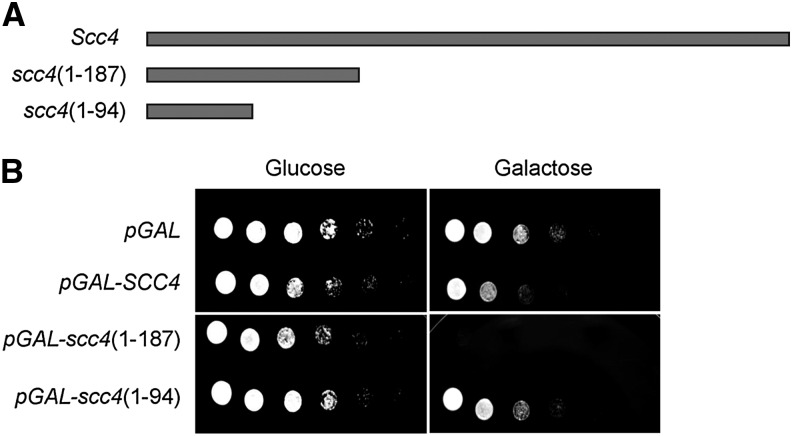
The 187-amino-acid N-terminal fragment of Scc4 is toxic when overexpressed. (A) The N-terminal truncations used in this study. (B) Strain YIO002 (*scc4-4*) cells carrying pRS406 (*pGAL URA3*), pIO014 (*pGAL-SCC4 URA3*), pMS018 (*pGAL-scc4-(1-187) CEN URA3*), or pMS019 (*pGAL-scc4-(1-94) CEN URA3*) were grown at 23° to saturation in SD-URA medium. Tenfold serial dilutions of each strain were plated on SD-URA plates containing either glucose or galactose and grown at 23°.

Our screen revealed that C-terminal truncations of Scc4 are toxic to cells. To identify the specific region associated with this phenotype, we performed a systematic truncation analysis. We constructed two CEN *URA3* plasmids that encode C-terminal *scc4* truncations under the control of a GAL promoter (pMS018 and pMS019) (Figure 2A). Plasmids were transformed into haploid strain YIO002, and their toxicity was tested on either glucose or galactose plates. We showed previously that overexpression of the C-terminal truncation alleles scc4 E374*, scc4 L465*, and scc4 Y551* completely inhibited cell growth (Figure 1C). Further reduction of the construct to the first 187 amino acids was still toxic. However, overexpression of a fragment containing the first 94 amino acids of Scc4 did not have a phenotypic effect on cell growth (Figure 2B). This finding suggests that an important domain is located between residues 94 and 187.

The crystal structure of Scc4 with the Scc2 fragment suggest that the domain located between amino acids 91 and 187 is most likely the interaction domain with Scc2 (Chao *et al.* 2015; Hinshaw *et al.* 2015). To test the interactions mediated by the Scc4 N-terminus, we expressed a 6 × His-tagged scc4 (1–187) fragment in bacteria. The Scc4-(1–187)-6 × His protein was soluble and we were able to purify it on nickel beads (data not shown). To test our hypothesis, we set up pull-down experiments of Scc2 from yeast extract. We repeated the experiment several times with different modifications of the protocol, but were unable to pull-down Scc2 with the Scc4 fragment (data not shown). The inability to pull-down Scc2 in these experiments is consistent with the cofolding model of Scc4 and Scc2 (Chao *et al.* 2015; Hinshaw *et al.* 2015).

### Single copy Scc4 RIDs do not support cell growth

To further dissect the molecular defect of Scc4-RIDs, we tagged both wild type *SCC4* and the *scc4*-RID mutant alleles with three copies of V5 epitopes (3V5) at their C-terminus, and cloned the genes under the control of the endogenous *SCC4* promoter. These alleles were integrated into the haploid strain YIO002 (*scc4-4*) at the *URA3* locus. The scc4-4 allele has been reported previously (Ciosk *et al.* 2000). It supports cell viability at 23° but not at 35°. The semi-permissive temperature is 30°. Cells are viable, but growth is slower compared to the isogenic wild-type strain (Figure S3A). At the restrictive temperature, cohesin is not loaded on the chromosomes at either the centromere or chromosome arms, and sister chromatid cohesion is lost (Figure 5B and Figure S3B). Inactivation of the scc4-4 allele at 35° at the G1 stage of the cell cycle does not block cell progression to G2/M (Figure 5C). In recent years, an AID system has been used as an alternative to ts alleles (Milutinovich *et al.* 2007; Eng *et al.* 2014; Orgil *et al.* 2015). We tried to construct such an SCC4-AID strain. However, the degradation of Scc4-AID is not efficient (data not shown). Therefore, the AID system is not feasible for studying the effects of the scc4 mutants isolated in this study. Consequently, from here on, we used the scc4-4 allele background to characterize the mutants in this work.

**Figure 3:**
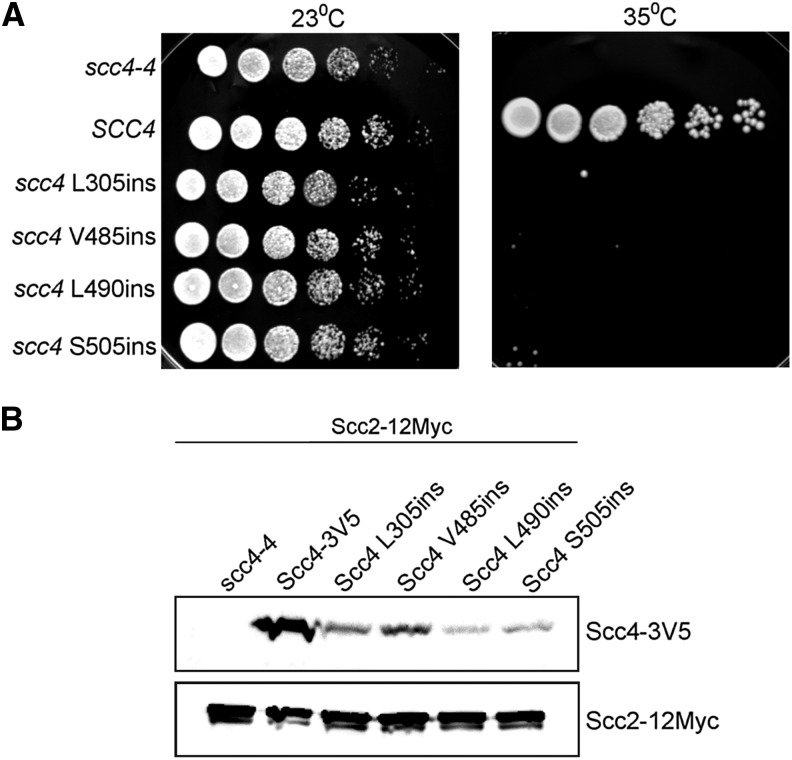
scc4 RID mutants do not support cell viability under the level of native expression. (A) Strains YIO002 (*scc4-4*), YMS1004 (*SCC4-3V5 scc4-4*), YMS1006 (*scc4L305ins-3V5 scc4-4*), YMS1007 (*scc4V485ins-3V5 scc4-4*), YMS1005 (*scc4L490ins-3V5 scc4-4*) and YMS1008 (*scc4-S505ins-3V5 scc4-4*) were grown to saturation in YPD media at 23°. Tenfold serial dilutions of each strain were plated on YPD plates and grown at either the permissive (23°) or restrictive (35°) temperature for *scc4-4*. (B) Protein extracts from strains YMS1003 (*SCC2-12Myc*), YMS1016 (*SCC2-12Myc SCC4-3V5*), YMS1017 (*SCC2-12Myc scc4-L305ins-3V5*), YMS1018 (*SCC2-12Myc scc4V485ins-3V5*), YMS1019 (*SCC2-12Myc scc4L490ins-3V5*), and YMS1020 (*SCC2-12Myc scc4- S505ins-3V5*) were analyzed by Western blot with antibodies against V5 (Scc4-3V5) and Myc (Scc2-12Myc). A representative blot is shown.

The parent strain alone, or bearing *SCC4-3V5*, *scc4-L305ins-3V5*, *scc4-V485ins-3V5*, *scc4-L490ins-3V5*, or *scc4-S505ins-3V5*, were grown to saturation, serially diluted on YPD plates, and incubated at 23° or 35°, the respective permissive and restrictive temperatures of the *scc4-4* allele (Figure 3A). At 23°, the *scc4-4* strain alone, and the scc4 alleles containing the insertions grew equally well. This suggests that when expressed in endogenous levels, scc4 mutants are recessive, in contrast to their dominant-negative phenotype, which is overexpressed (Figure 1A). At 35°, the *scc4-4* strain alone and all strains containing a RID allele failed to grow, whereas the *SCC4-3V5 scc4-4* strain was viable.

To verify expression of the tagged alleles *in vivo*, we analyzed protein extract prepared from these strains by Western blot with antibodies against the V5 tag. In all strains, we identified a single band of the expected molecular weight that was absent in the untagged parent, indicating that the protein is expressed. However, we noticed a reduction in the steady-state level of the mutant proteins compared with the wild-type protein. No similar reduction was observed in Scc2-12Myc in the same cells (Figure 3B), suggesting that the instability is not associated with the V5-tag. Similar levels of protein were detected when cells were grown at either 23°, 30°, or 35° suggesting that the protein level is not temperature dependent (Figure S4). The reduction in scc4 mutants cannot be explained by the addition of the tag itself because it was not observed in the wild-type protein. Therefore, we concluded that the instability is associated with the mutation in the proteins. However, the detectable steady-state levels of the proteins should be sufficient for molecular characterization of the associated phenotypes.

The inability of the scc4 -RIDs to support cell viability can be explained by two models. In the first model, when expressed to endogenous levels, the mutant alleles are recessive, and do not support cell viability as a sole copy. Alternatively, failure of the RID mutants to support viability is the result of protein instability. If the first model is correct, we expect that some of the molecular interactions will be maintained. However, in the other case, the mutants will not be able to interact with Scc2 or cohesin. To discriminate between these models, we explored the integrity of the loader.

### Scc4 RIDs interact with Scc2

**Figure 4:**
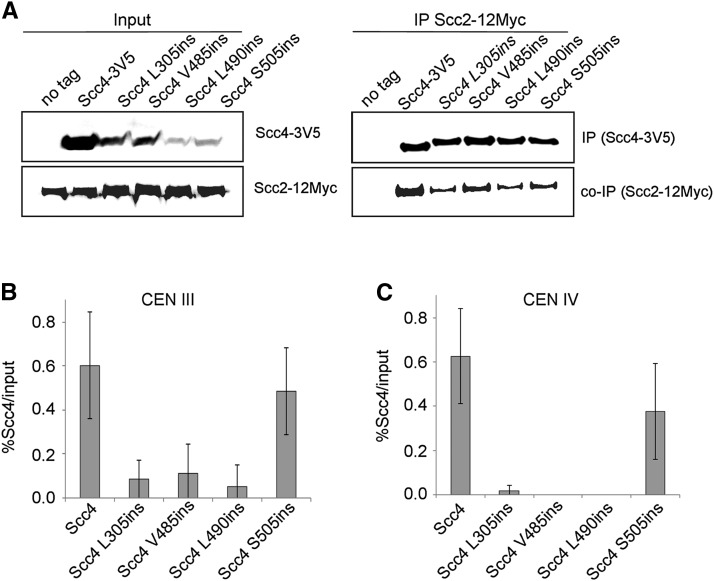
RID mutations do not affect loader integrity. (A) Haploid YMS1003 (*SCC2-12Myc*), YMS1016 (*SCC2-12Myc SCC4-3V5*), YMS1017 (*SCC2-12Myc scc4-L305ins-3V5*), YMS1018 (*SCC2-12Myc scc4V485ins-3V5*), YMS1019 (*SCC2-12Myc scc4L490ins-3V5*), and YMS1020 (*SCC2-12Myc scc4S505ins-3V5*) were grown to midlog phase in YPD medium at 23°. Cells were lysed, and the protein extracts were subjected to IP against the V5 tag (Scc4). Precipitated Scc2 was analyzed by Western blot using antibodies against Myc. (B) Strains YIO002 (*scc4-4*), YMS1004 (*SCC4-3V5 scc4-4*), YMS1006 (*scc4L305ins-3V5 scc4-4*), YMS1007 (*scc4V485ins-3V5 scc4-4*), YMS1005 (*scc4L490ins-3V5 scc4-4*), and YMS1008 (*scc4S505ins-3V5 scc4-4*) were processed for ChIP analysis. V5 tagged Scc4 proteins were immunoprecipitated (*n* = 3). Precipitated DNA was analyzed by quantitative PCR. Analysis of centromere III is shown. (C) Analysis of centromere IV is shown.

We examined the ability of *scc4-L305ins-3V5*, *scc4-V485ins-3V5*, *scc4-L490ins-3V5*, and *scc4-*S*505ins-3V5* to coimmunoprecipitate the loader subunit Scc2. Extracts from YMS1003 (*SCC2-12Myc*), YMS1016 (*SCC2-12Myc SCC4-3V5*), YMS1017 (*SCC2-12Myc scc4-L305ins-3V5*), YMS1018 (*SCC2-12Myc scc4-V485ins-3V5*), YMS1019 (*SCC2-12Myc scc4L490ins-3V5*), and YMS1020 (*SCC2-12Myc scc4-S505ins-3V5*) cells were prepared. Cells were grown to midlog phase, and Scc4 was precipitated by using anti-V5 antibodies (*Materials and Methods*). Scc2-12Myc coprecipitated with wild-type Scc4. However, the coprecipitation of Scc2-12Myc with scc4-L305ins-3V5, scc4-V485ins-3V5, scc4-L490ins-3V5, and scc4-*S*505ins-3V5 was similarly reduced to about 50% of the wild-type level (Figure 4A). The reduction in co-IP levels cannot be explained by the reduced levels of Scc4-3V5 in the cells since protein levels in the IP were similar. This suggests that the antibody was the limiting factor in the experiment. As such, scc4 -RIDs appear to maintain their ability to interact with Scc2, while stability of the dimer is reduced. If the loader is formed in the context of the mutants, the activity and effect on cohesin can be studied further.

We tested the ability of the Scc2/scc4-RID complex to bind chromatin by ChIP. Strains YMS1004 (*SCC4-3V5*), YMS1005 (*scc4-L490ins-3V5*), YMS1006 (*scc4-L305ins-3V5*), YMS1007 (*scc4-V485ins-3V5*), and YMS1008 (*scc4-S505ins-3V5*) were arrested in the G2/M phase, and processed for ChIP using anti-V5 antibodies. Then, Scc4 binding to chromosome III and IV centromeres was assessed using quantitative PCR analysis (Figure 4, B and C). No binding was detected in the presence of scc4-V485ins-3V5, scc4-L305ins-3V5, and scc4-L490ins-3V5, while 60% of wild-type binding was found for scc4-S505ins-3V5. These results suggest that the regions flanking L305 and L490 are essential for stable binding of the loader to chromatin. The fact that Scc4 RIDs interact with Scc2 supports the supposition that the activity of the Scc2/Scc4 dimer requires an intimate interaction between the two proteins.

### Scc4 RIDs do not support sister chromatid cohesion

**Figure 5:**
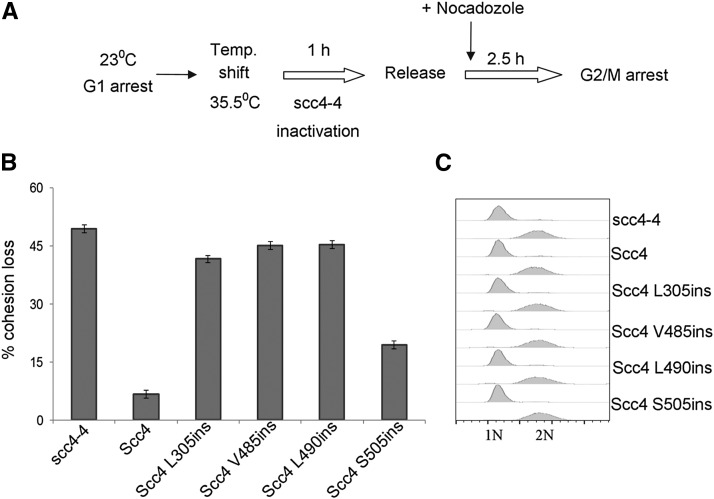
scc4-RIDs do not support sister chromatin cohesion. (A) Flowchart of the experimental design to score sister chromatid cohesion. (B) Strains YIO002 (*scc4-4*), YMS1011 (*SCC4-3V5 scc4-4*), YMS1013 (*scc4L305ins-3V5 scc4-4*), YMS1014 (*scc4V485ins-3V5 scc4-4*), YMS1012 (*scc4L490ins-3V5 scc4-4*), and YMS1015 (*scc4S505ins-3V5 scc4-4*) were processed for cohesion assay as shown in A (*n* = 3). (C) Flow cytometry analysis of strains from the cohesion assay.

Scc4 RIDs and Scc2 dimerize but the mutant loader is unable to support cell viability. In this case, we expect that the nonfunctional single copy mutant Scc4 will not support cohesion in mitotic cells. To test this possibility, we measured cohesion by the GFP dot assay (*Materials and Methods*). Strains YMS1010 (*scc4-4*), YMS1011 (*SCC4-3V5 scc4-4*), YMS1012 (*scc4-L490ins-3V5 scc4-4*), YMS1013 (*scc4-L305ins-3V5 scc4-4*), YMS1014 (*scc4-V485ins-3V5 scc4-4*), and YMS1015 (*scc4-S505ins-3V5 scc4-4*) were arrested in G1 phase, shifted to the nonpermissive temperature for *scc4-4* (35.5°), then released from G1 into medium containing nocodazole at 35.5°, to rearrest cells in G2/M (Figure 5A). About 95% of *SCC4-3V5 scc4-4* control cells had a single GFP dot, indicative of robust cohesion. In contrast, in *scc4-4* cells, and in *scc4-L305ins-3V5 scc4-4*, *scc4-V485ins-3V5 scc4-4*, and *scc4-L490ins-3V5 scc4-4* cells, ∼50% of two GFP spots were detected in cells arrested at the G2/M phase, indicative of a severe defect in cohesion (Figure 5B). The cohesion defect in *scc4-S505ins-3V5 scc4-4* cells was milder, and consistent with the milder growth phenotype when this allele was overexpressed (Figure 1B). Flow cytometry of the cells indicated that, under these conditions, cells progress through S phase and were arrested in G2/M. This suggests that the two spots are not the result of a defect in DNA replication (Figure 5C). An interaction between Scc2 and scc4 RIDs seems to be insufficient for a functional loader.

### Scc4 mutants inhibit cohesin loading

**Figure 6:**
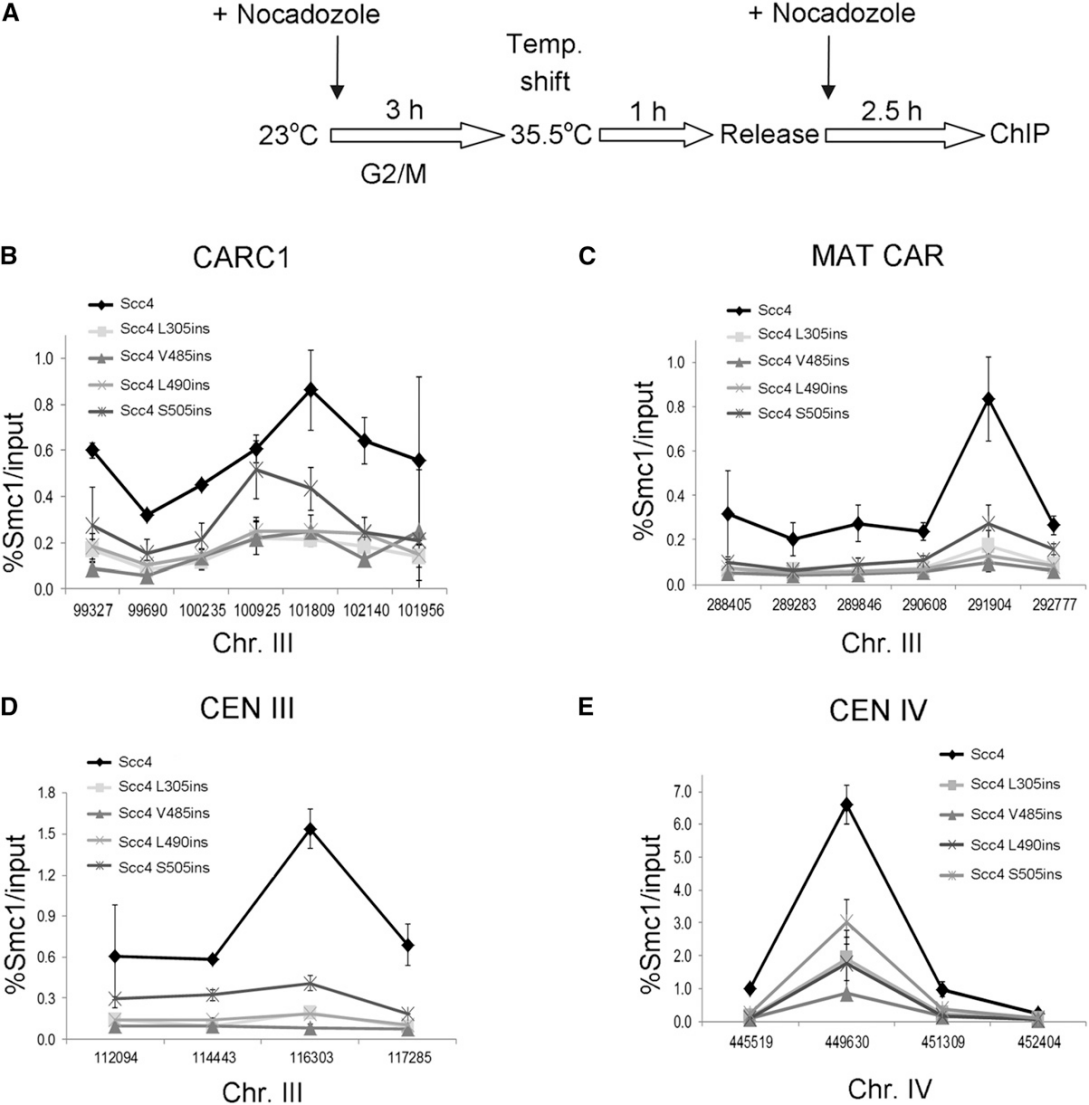
Scc4 is required for cohesin loading. (A) Flowchart of the experimental design. (B) Strains YIO002 (*scc4-4*), YMS1004 (*SCC43V5 scc4-4*), YMS1006 (*scc4L305ins-3V5 scc4-4*), YMS1007 (*scc4V485ins-3V5 scc4-4*), YMS1005 (*scc4L490ins-3V5 scc4-4*), and YMS1008 (*scc4S505ins-3V5 scc4-4*) were processed for ChIP analysis. Scc4, V5 tagged proteins were immunoprecipitated. Precipitated chromosome III CARC1 DNA was analyzed by quantitative PCR (*n* = 3). (C) Analysis of the MAT CAR on chromosome III. (D) Analysis of centromere III. (E) Analysis of centromere IV.

Given the importance of Scc4 to cohesin loading, we predicted that the Scc4-RIDs/Scc2 complexes are unable to load cohesin onto the chromosomes. To test this hypothesis we performed ChIP from strain YIO002 (*scc4-4)* carrying the cohesin subunit *SMC1* tagged with six copies of hemagglutinin epitope (*SMC1-6HA*), and *scc4-L305ins-3V5*, *scc4-V485ins-3V5*, *scc4-L490ins-3V5*, or *scc4-S505ins-3V5*. Strains were grown at permissive temperature, arrested in the G2/M phase at 23°, shifted to 35.5° to inactivate *scc4-4*, then released back to the cell cycle at 35.5° and rearrested in G2/M. The arrested cells were processed for ChIP using anti-HA antibodies (Figure 6A). Smc1 binding to the known cohesin associated regions CARC1 and MAT CAR on chromosome III, and centromeres III and IV was measured using quantitative PCR (Figure 6, B–E). In cells carrying the *SCC4* wild-type allele, Smc1 was detected, as expected. In the presence of *scc4-L305ins-3V5*, *scc4-V485ins-3V5*, and *scc4-L490ins-3V5*, Smc1 was not detected in two arm cohesin associated regions (CARs), nor in centromeres III and IV. This suggests that mutant scc4 are unable to load cohesin onto chromosome arms. Cohesin loading in the presence of scc4-S505ins was lower than in the wild type but greater than background levels. This supports previous data.

### The interaction of Scc4 with cohesin depends on Scc2

**Figure 7:**
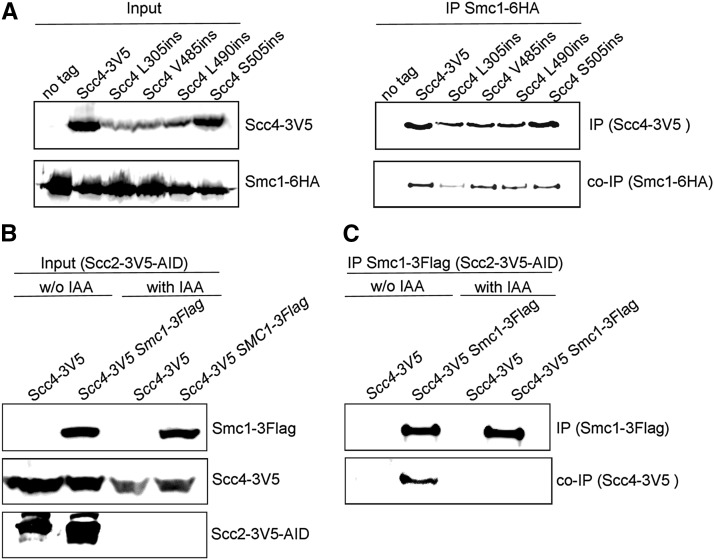
RID mutations do not affect the interaction with cohesin. (A) Haploid YIO002 (*SMC1-6HA*), YMS1004 (*SMC1-6HA SCC4-3V5*), YMS1006 (*SMC1-6HA scc4-L305ins-3V5*), YMS1007 (*SMC1-6HA scc4-V485ins-3V5*), YMS1005 (*SMC1-6HA scc4-L490ins-3V5*) or YMS1008 (*SMC1-6HA scc4-S505ins-3V5*) were grown to midlog phase in YPD media at 23°. Cells were processed and subjected to immunoprecipitation against the V5 tag (Scc4). Precipitated proteins were analyzed by Western blot using antibodies against V5 (IP) and HA (co-IP). (B) Haploid YMS1021 (*SCC2-AID-3V5 SCC4-3V5*), and YMS1022 (*SCC2-AID-3V5 SCC4-3V5 SMC1-3FLAG*) cells were grown to midlog phase in YPD media at 23°. The cultures were divided into two, and cells were grown for an additional 1.5 hr with or without the addition of 1 mM IAA. Cells were lysed and the extract was analyzed by Western blot with anti-V5 and anti-FLAG antibodies. (C) The protein extracts from A were subjected to immunoprecipitation against the FLAG tag (Smc1). Precipitated Scc4 was analyzed by Western blot using antibodies against V5.

Scc4 interacts with Scc2 to form the loading dimer and the dimer interacts with cohesin. To test the ability of the scc4 mutants to interact with cohesin, we used cell extracts from the strains containing the Scc4-RID-3V5 and the cohesin subunit Smc1 tagged with six copies of hemagglutinin epitope (SMC1-6HA): YIO002 *(SMC1-6HA)*, YMS1004 (*SMC1-6HA SCC4-3V5*), YMS1005 (*SMC1-6HA scc4-L490ins-3V5*), YMS1006 (*SMC1-6HA scc4-L305ins-3V5*), YMS1007 (*SMC1-6HA scc4-V485ins-3V5*), and YMS1008 (*SMC1-6HA scc4-S505ins-3V*). The cells were grown to midlog phase, lysed and subjected to immunoprecipitation with anti-V5 antibodies. The co-IP of Smc1-6HA was at wild type levels with Scc4, *s*cc4-V485ins-3V5, scc4-L490ins-3V5, and scc4-S505ins-3V5, but reduced with scc4-L305ins-3V5 (Figure 7A).

At this stage, we sought to test if Scc4 interacts directly with cohesin. To explore this possibility, we built strains YMS1021 and YMS1022, in which Scc2 can be depleted *in vivo* by an AID system (Figure 7B). Strain YMS1021 contains Scc4 tagged with 3V5, while YMS1022 contains Smc1 and Scc4 tagged with 3FLAG and 3V5 epitopes, respectively. We immunoprecipitated Smc1 from untreated cells, or from cells treated with 1 mM IAA to deplete Scc2, and tested the coprecipitation of Scc4. As expected, Scc4 was detected when Scc2 was present. However, we could not detect Scc4 when Scc2 was depleted (Figure 7C). This shows that Scc2 is indispensable for the Scc4–cohesin interaction.

To further dissect the domains that mediate the molecular interactions of Scc4, we introduced missense mutations in residues that are located in the insertion region, and that are evolutionarily conserved. The mutant alleles were transformed into *scc4-4* cells, and tested for their ability to support cell growth by a semiquantitative assay. None of the point mutations we inserted mimicked the phenotype of the insertion alleles, and no effect on cell growth was observed (Figure S5).

## Discussion

Analysis of Scc4 reveals a complex pattern of molecular interactions. We used a genetic approach to isolate mutants that highlighted specific properties of the protein. The RID screen revealed four functional domains in the protein. This work demonstrates the power of the RID genetic screen approach in identifying functional domains in proteins that mediate complex interactions. We showed that the regions we identified in the protein affect cohesin loading onto chromosomes and cohesion but they have a minor effect on the binding of Scc4 to its loading-dimer partner Scc2, and the physical interaction with cohesin.

The genetic screen we describe is based on the dominant negative nature of the alleles when overexpressed in the presence of a functional allele. Ectopic overexpression of SCC4 in yeast cells does not affect cell growth (Figure 1). Random insertion of a short sequence into the overexpressed SCC4 plasmid can lead to several outcomes. First, an insertion may have no effect on the expression of SCC4 if is localized to a nonfunctional region of the vector. In this case, ectopic SCC4 expression will not be affected. Second, the insertion may affect expression by abolishing the gene promoter. In this case, the ectopic SCC4 will not be expressed. However, cell viability is maintained by the native SCC4 allele and growth will not be affected. Third, the short sequence is inserted in the coding sequence, thus destroying protein activity or stability. As before, these possibilities will have no phenotypic outcome because cells contain a second SCC4 allele. Regarding the last possibility, insertion is again in the coding sequence. However, in this scenario, the mutated protein is partially active. This can happen if the protein has several functions that are mediated by distinct domains, *e.g.*, two separate protein–protein interaction domains. The insertion locally destroys one domain without affecting other domains of the protein. This partially active protein can form partial inactive complexes. When the partial active allele is overexpressed, it competes with the native allele, and titers out essential interactors that are required for the formation of an active complex. In contrast to the other possibilities, this scenario, known as a dominant negative effect, will have a phenotypic outcome, which, in the case of SCC4, is loss of cell viability and cohesin loading. The dominant negative effect can be detected in multi-functional, multi-domain proteins, and is apparent only when the effect of the mutation is limited to a defined domain, with no global effects on protein stability or function. Scc4 is a multifunctional protein. Three molecular interactions have been associated with Scc4: Scc2, cohesin, and chromatin. The isolation of dominant negative mutants in this study suggests that these functions are mediated by distinct domains that can be disrupted locally, while the other functions of Scc4 are maintained.

The dominant negative effect of the overexpressed scc4 alleles demonstrates that the insertion mutants are undoubtedly partly active. However, when RID mutants were expressed as a single copy, their levels were lower than those of the wild type proteins. This may be due to lower expression, or to instability of the proteins. The activities of growth inhibition, failure to load cohesin, and exclusion of Scc4 from the chromatin described herein can be explained by the partial activity of the mutant proteins, as a result of disruption of a functional domain. However, an alternative explanation is the reduced levels of the protein. The first explanation is more likely for several reasons. First, we constructed a library in which transposons were inserted every three amino acids, yet we isolated only five mutants that are dominant negative when overexpressed. In the second scenario, we would expect to find a large number of loss-of-function mutants. Second, the dominant-negative property of the mutants indicates, without any doubt, that the overexpressed mutant proteins are partially active. Third, while the cell growth phenotype can be associated with loss-of function, protein–protein interaction of mutant scc4 with both Scc2 and Smc1 are maintained. This suggests that protein complexes are formed but are inactive. Fourth, our analysis identified mutations in the helix bundle, which was previously identified as an important region for centromeric cohesin loading (Fernius *et al.* 2013). The identification of one important domain increases confidence that the other domains identified by the screen are of functional importance. Finally, the stability of the S505ins mutant is similar to that of the other mutants. However, this mutant reveals phenotypes that are different from the other mutants, indicating that the expression level is sufficient to support the loader’s functions. Altogether, the results of the screen provide reliable information on the structure–function relationship of Scc4.

Previous studies paid little attention to Scc4, and even less to its specific functions in loader activity. Most recently, the crystal structure of Scc4 in the complex with the N-terminus of Scc2 was solved (Chao *et al.* 2015; Hinshaw *et al.* 2015). Scc4 is wrapped around Scc2, which is mostly unfolded. The RID mutants we examined were located at different regions of Scc4, but they reduced the interaction affinity with Scc2 in a similar manner. Interestingly, the RID mutations in Scc4 are located in proximity to small regions in which the secondary structure of Scc2 is defined. The integration of the structural, genetic, and biochemical data suggests that Scc4 forms a tight complex with Scc2, and that a compact structure is required for loading activity.

We and others have shown that Scc2/Scc4 has multiple interactions with cohesin (Murayama and Uhlmann 2014, 2015; Orgil *et al.* 2015). Scc2 is a required component of the loader, which mediates the interaction with cohesin. The results of this study elucidate the contribution of Scc4 to the binding. We showed that the essential Scc2 binding domain is located at the N-terminus of Scc4. Our results also suggest that Scc2 is essential for cohesin binding. Interestingly, scc4 -L305ins demonstrated reduced binding to the Smc1. L305 is located at the conserved patch of Scc4 (Hinshaw *et al.* 2015). In contrast to a previous study showing that mutations in this region reduce cohesin binding to centromeres but not chromosome arms, the L305ins mutant failed to load cohesin in both regions (Hinshaw *et al.* 2015). The results imply that this region is important but not essential for the interaction with cohesin.

Mutations in Scc2 are the major cause of CdLS (Krantz *et al.* 2004). However, no mutation has been reported in Scc4 in association with a human disorder. Three possible explanations arise: one is that mutations in SCC4 have not yet been identified but will be identified in human patients in the future. The second possibility is that Scc4 is not essential in humans. Studies have shown that RNA knockdown of SCC4 in HeLa cells causes a chromosome segregation defect, and that knockout SCC4 mice showed a more severe phenotype than did SCC2 knockout mice (Seitan *et al.* 2006; Smith *et al.* 2014). Third, inactivation of Scc4 by a mutation is lethal. This raises the question as to the functional difference between Scc2 and Scc4. Scc2 may play a role in transcription that is independent of Scc4 (Dorsett *et al.* 2005; Lin *et al.* 2011; Lopez-Serra *et al.* 2014; Zakari and Gerton 2015). The instability of our mutants also suggests that the tolerance of Scc4 to mutation may be low, and such changes may lead to cell death. Our work contributes to deciphering the role of Scc4, and sheds new light on the mechanisms of cohesin loading and genome stability.

## Supplementary Material

Supplemental Material
