# Supplementary material for: Identification of Functional Domains in the Cohesin Loader Subunit Scc4 by a Random Insertion/Dominant Negative Screen
Source: G3 (Bethesda). 2016 Jun 7;6(8):2655–63. doi: 10.1534/g3.116.031674 (PMC4978918; doi:10.1534/g3.116.031674)
Supplement: Supplemental Material [file supp_6_8_2655__index.html]

Identification of Functional Domains in the Cohesin Loader Subunit Scc4 by a Random Insertion/Dominant Negative Screen — Supplemental Material 

# Identification of Functional Domains in the Cohesin Loader Subunit Scc4 by a Random Insertion/Dominant Negative Screen

## Supplemental Material for Shwartz, Matityahu, and Onn, 2016

**Files in this Data Supplement:**

- Table S1 - Yeast Strains. (.pdf, 74 KB)
- Table S2 - Plasmids. (.pdf, 14 KB)
- Table S3 - Primers. (.pdf, 67 KB)
- Figure S1 - Genetic screen to isolate scc4 RID mutants. Flowchart of the experimental design to identify *SCC4* dominant negative mutants. B. *E. coli* cells were transformed with plasmids from stage 2 in A. At this stage plasmids contain a resistance gene to ampicillin and kanamycin. Colonies on ampicillin and kanamycin plates were 2x10^3^ CFU/ml and 6x10^2^ CFU/ml, respectively. C. *E. coli* cells were transformed with plasmids from stage 4. The plasmids contain a resistance gene to ampicillin but most of them lost their kanamycin resistance gene. 3x10^2^ CFU/ml were counted on the ampicillin plates, while 0.9 CFU/ml were formed on the kanamycin plates. (.tif, 887 KB)
- Figure S2 - Localization of the insertion domains on Scc4 structure. The location of the functional domains on the Scc2/4 structure (PDB 4XDN) are indicated. Scc2 is shown in purple. Scc4 is in yellow. The N' terminal is shown in orange and the regions of the insertions are colored in red. (.tif, 667 KB)
- Figure S3 - The scc4-4 allele does not support cohesin loading at restrictive temperature. A. YIO002 (*scc4-4*) and YMS1004 (*SCC43V5 scc4-4*) were streaked on YPD plates that were incubated at 23°C, 30°C or 35°C. B. YIO002 (*SMC1-6HA* scc4-4) and YMS1004 (*SMC1-6HA SCC4-3V5*), were grown and processed for ChIP with antibody against HA as described in Figure 6. Smc1 binding was determined on the centromere of chromosome III, and at the MAT CAR on chromosome III arm. (.tif, 858 KB)
- Figure S4 - Similar levels of Scc4-RIDs at different temperatures. Haploid YMS1003 (*SCC2-12Myc*), YMS1016 (*SCC2-12Myc SCC4-3V5*), YMS1017 (*SCC2-12Myc scc4-L305ins-3V5*), YMS1018 (*SCC2-12Myc scc4-V485ins-3V5*), YMS1019 (*SCC2-12Myc scc4- L490ins-3V5*) and YMS1020 (*SCC2-12Myc scc4- S505ins-3V5*) were grown to mid-log phase in YPD media at 23°C, and then, transferred to 23°C, 30°C or 35°C for additional 2h. Cells were lysed and the extract was analyzed by Western blot with anti-V5 (Scc4) and anti-Myc (Scc2) antibodies. (.tif, 460 KB)
- Figure S5 - Site directed mutagenesis of SCC4. Multiple sequence alignment of SCC4 generated by ClustalW. A. The alignment region corresponding to *S. cerevisiae* Scc4 amino acids L306 and L307 is shown B. The alignment region corresponding to *S. cerevisiae* Scc4 amino acids W489 and L490 is shown. C. Strains YIO002 (*scc4-4*), YMS1004 (*SCC43V5 scc4-4*), YMS1023 (*scc4* L305E-3V5 *scc4-4*), YMS1024 (*scc4* L306E-3V5 *scc4-4*), YMS1025 (*scc4* W489A-3V5 *scc4-4*) and YMS1026 (*scc4* L490E-3V5 *scc4-4*) were grown to saturation in YPD media. Tenfold serial dilutions of each strain were plated on YPD plates and grown at either the permissive (23°) or restrictive (35°) temperature for *scc4-4*. (.tif, 1,750 KB)
