# Supplementary material for: Identification of Functional Domains in the Cohesin Loader Subunit Scc4 by a Random Insertion/Dominant Negative Screen
Source: G3 (Bethesda). 2016 Jun 7;6(8):2655–63. doi: 10.1534/g3.116.031674 (PMC4978918; doi:10.1534/g3.116.031674)
Supplement: Supplemental Material [file supp_g3.116.031674_TableS1.pdf]

Supplementary Table S1. Yeast Strains

Strain	Genotype
YIO002	*MATa scc4Δ::HIS3 scc4-4::LEU2 SCC3-18MYC::TRP1 SMC1-6HA::HIS3 pep4Δ::LEU2 ade2*
YMS1004	*MATa scc4Δ::HIS3 scc4-4::LEU2 SCC3-18MYC::TRP1 SMC1-6HA::HIS3 pep4Δ::LEU2 ade2 ura3::SCC4-3V5:URA3*
YMS1005	*MATa scc4Δ::HIS3 scc4-4::LEU2 SCC3-18MYC::TRP1 SMC1-6HA::HIS3 pep4Δ::LEU2 ade2 ura3::scc4-L490ins-3V5:URA3*
YMS1006	*MATa scc4Δ::HIS3 scc4-4::LEU2 SCC3-18MYC::TRP1 SMC1-6HA::HIS3 pep4Δ::LEU2 ade2 ura3::scc4-L305ins-3V5:URA3*
YMS1007	*MATa scc4Δ::HIS3 scc4-4::LEU2 SCC3-18MYC::TRP1 SMC1-6HA::HIS3 pep4Δ::LEU2 ade2 ura3::scc4-V485ins-3V5:URA3*
YMS1008	*MATa scc4Δ::HIS3 scc4-4::LEU2 SCC3-18MYC::TRP1 SMC1-6HA::HIS3 pep4Δ::LEU2 ade2 ura3::scc4-S505ins-3V5:URA3*
YMS1010	*MATa trp1-1 leu2-3,112 ura3-52 his3-11,15 GAL+scc4-4 cloNAT lys4::LacO GFP-LacI-HIS3*
YMS1011	*MATa trp1-1 leu2-3,112 ura3-52 his3-11,15 GAL+ scc4-4 cloNAT lys4::LacO GFP-LacI-HIS3 SCC4-3V5 URA3*
YMS1012	*MATa trp1-1 leu2-3,112 ura3-52 his3-11,15 GAL+ scc4-4 cloNAT lys4::LacO GFP-LacI-HIS3 ura3::scc4-L490ins-3V5:URA3*
YMS1013	*MATa trp1-1 leu2-3,112 ura3-52 his3-11,15 GAL+scc4-4 cloNAT lys4::LacO GFP-LacI-HIS3 ura3::scc4-L305ins-3V5:URA3*
YMS1014	*MATa trp1-1 leu2-3,112 ura3-52 his3-11,15 GAL+ scc4ts cloNAT lys4::LacO GFP-LacI-HIS3 ura3::scc4-V485ins-3V5:URA3*
YMS1015	*MATa trp1-1 leu2-3,112 ura3-52 his3-11,15 GAL+ scc4ts cloNAT lys4::LacO GFP-LacI-HIS3 ura3::scc4-S505ins-3V5:URA3*
YMS1003	*MATa pep4Δ::g418::TRP1 trp1-1 leu2-3,112 ura3-52 his3-11,15 bar1 GAL+ his3::SCC2-12MYC:HIS3 MX*
YMS1016	*MATa pep4Δ::g418::TRP1 trp1-1 leu2-3,112 ura3-52 his3-11,15 bar1 GAL+ his3::SCC2-12MYC:HIS3 MX, ura3::SCC4-3V5:URA3*
YMS1017	*MATa pep4Δ::g418::TRP1 trp1-1 leu2-3,112 ura3-52 his3-11,15 bar1 GAL+ his3::SCC2-12MYC:HIS3 MX, ura3::scc4-L305ins-3V5:URA3*

Strain	Genotype
YMS1018	*MATa pep4Δ::g418::TRP1 trp1-1 leu2-3,112 ura3-52 his3-11,15 bar1 GAL+ his3::SCC2-12MYC:HIS3 MX, ura3::scc4-V485ins-3V5:URA3*
YMS1019	*MATa pep4Δ::g418::TRP1 trp1-1 leu2-3,112 ura3-52 his3-11,15 bar1 GAL+ his3::SCC2-12MYC:HIS3 MX, ura3::scc4-L490ins-3V5:URA3*
YMS1020	*MATa pep4Δ::g418::TRP1 trp1-1 leu2-3,112 ura3-52 his3-11,15 bar1 GAL+ his3::SCC2-12MYC:HIS3 MX, ura3::scc4-S505ins-3V5:URA3*
YMS1021	*MATa SCC2-3V5-AID2-G418 TIR1-CaTRP1 LacO(DK)-NAT::lys4 leu2-3,112 GAL+ pHIS3-GFPLacI-HIS3:his3-11,15 bar1 ura3-52 ura3::SCC4-3V5:URA3*
YMS1022	*MATa SCC2-3V5-AID2-G418 TIR1-CaTRP1 LacO(DK)-NAT::lys4 leu2-3,112 GAL+ pHIS3-GFPLacI-HIS3:his3-11,15 bar1 ura3-52 ura3::SCC4-3V5:URA3, leu2::SMC1-3Flag:LEU2*
YMS1023	*MATa scc4Δ::HIS3 scc4-4::LEU2 SCC3-18MYC::TRP1 SMC1-6HA::HIS3 pep4Δ::LEU2 ade2 ura3::scc4-L306E-3V5:URA3*
YMS1024	*MATa scc4Δ::HIS3 scc4-4::LEU2 SCC3-18MYC::TRP1 SMC1-6HA::HIS3 pep4Δ::LEU2 ade2 ura3::scc4-L307E-3V5:URA3*
YMS1025	*MATa scc4Δ::HIS3 scc4-4::LEU2 SCC3-18MYC::TRP1 SMC1-6HA::HIS3 pep4Δ::LEU2 ade2 ura3::scc4-W490A-3V5:URA3*
YMS1026	*MATa scc4Δ::HIS3 scc4-4::LEU2 SCC3-18MYC::TRP1 SMC1-6HA::HIS3 pep4Δ::LEU2 ade2 ura3::scc4-L491E-3V5:URA3*
