# Supplementary material for: Identification of Functional Domains in the Cohesin Loader Subunit Scc4 by a Random Insertion/Dominant Negative Screen
Source: G3 (Bethesda). 2016 Jun 7;6(8):2655–63. doi: 10.1534/g3.116.031674 (PMC4978918; doi:10.1534/g3.116.031674)
Supplement: Supplemental Material [file supp_g3.116.031674_TableS2.pdf]

Supplementary Table S2. Plasmids

Plasmid	Description
pIO014	pGAL-*SCC4 CEN URA3*
pMS011	pGAL-*SCC4-L305ins CEN URA3*
pMS012	pGAL-*SCC4-V485ins CEN URA3*
pMS013	pGAL-*SCC4-L490ins CEN URA3*
pMS014	pGAL-*SCC4-S505ins CEN URA3*
pMS015	pGAL-*SCC4-E374* CEN URA3*
pMS016	pGAL-*SCC4-L465* CEN URA3*
pMS017	pGAL-*SCC4-Y551* CEN URA3*
pMS018	pGAL-*Scc4(1-187) CEN URA3*
pMS019	pGAL-*Scc4(1-94) CEN URA3*
pMS2	*SCC4-3V5 URA3*
pMS024	*scc4-L305ins-3V5 URA3*
pMS025	*scc4-V485ins-3V5 URA3*
pMS026	*scc4-L490ins-3V5 URA3*
pMS027	*scc4-S505ins-3V5 URA3*

pMS028	*scc4-L306E-3V5 URA3*
pMS029	*scc4-L307E-3V5 URA3*
pMS030	*scc4-W490A-3V5 URA3*
pMS031	*scc4-L491E-3V5 URA3*
pMS041	pET28a-*Scc4(1-187)* Kan^r
