# Supplementary material for: Identification of Functional Domains in the Cohesin Loader Subunit Scc4 by a Random Insertion/Dominant Negative Screen
Source: G3 (Bethesda). 2016 Jun 7;6(8):2655–63. doi: 10.1534/g3.116.031674 (PMC4978918; doi:10.1534/g3.116.031674)
Supplement: Supplemental Material [file supp_g3.116.031674_TableS3.pdf]

Supplementary Table S3.   Primers

Location		Forward	Reverse
Chr. III (CARC1)	99327	AGCGGATCAATCCACAAAGC	TGCTGTAGTCACCTCAGCAAG
	99690	AAAGGTGCCCCAAGAAAAGG	AGCACTTTACTCGCTTGTGG
	100235	ATGCCAAGGCGGAAAGAATG	TGGGGGCTTCTCGATTTTTG
	100925	ATGAGAAAGAGGGGTTCCTTCG	GGCGTCAATGCTTTAGTTCTCC
	101809	ACTTTGGTTTTCCGGTGTGC	CCAGCGATGAGATGCGAAAAG
	102140	ATGGTTCGGTTGGTGCTTAG	ACGCGGAATTGAAACCACAG
	101956	TCGCTTTTCGCATCTCATCG	AGCGGGCGGGTTATAAATAAC
Chr. III (MAT CAR)	288405	TCGTCTCCTAGAACTCCACTTAC	GGCCAATTCTGGCAAACTTATT
	289283	CCAGTGCTGTAAGTGTCTCTC	GCCTTGACATATCCGGGTAATA
	289846	TGTAGGTGCAGAAAGAGAAAGT	CCAAGGCATAATGTGTGAAGTG
	290608	GTTGTATTTCTTTCCGTCTCACATT	CAAGACTCACCTTCCAGTACATAG
	291904	AGAGTTGGTAGAGTTCCTTGTTG	ACTACCGGATTAGAGGTTTGTTATT
	292777	CCCGTCCAAGTTATGAGCTTAAT	CAGGAGTACCTGCGCTTATTC
Chr. III (centromere)	112094	CCGAAGGCTGGTATGTGATT	CCAGCTTCCAATTCGTTGTTT
	114443	GATCAGCGCCAAACAATATGG	AACTTCCACCAGTAAACGTTTC
	116303	TGAGTCTTCGTCAGTGCATATT	CACTTTCATGTTTGGACGTTTCT
	117285	ACCAAGGAGAGAAGCAAGTTAC	ATGTCTAGCAACGCCATTCA
Chr. IV (centromere)	445519	GGTTGGGATCTAGGGATTAC	TGATTGATTCACCTAGCCTT
	449630	ACACGAGCCAGAAATAGTAAC	TGATTATAAGCATGTGACCTTT
	451309	GGAATACCGAGACCGTTAG	ACAGCCCCCATTCTTG
	452404	CTTGGGTCTGTTGGGG	ATACAAGCCAAGGACCG

Supplementary Table S2.Primers used for site directed mutagenesis

Mutation	Forward primer	Reverse primer
L306E	gaacatactgGAattattgcaaagtgt	tttaattctttgtagtggaaaatcataggc
L307E	gaacatactgttaGAattgcaaagtgt	
W490A	aacgatcctaGCgcttttcagccatttcg	actgtgcactcccatatggggttg
L491E	aacgatcctatggGAAttcagccatttcg	
