# Supplementary figures and images for: Identification of Functional Domains in the Cohesin Loader Subunit Scc4 by a Random Insertion/Dominant Negative Screen
Source: G3 (Bethesda). 2016 Jun 7;6(8):2655–63. doi: 10.1534/g3.116.031674 (PMC4978918; doi:10.1534/g3.116.031674)

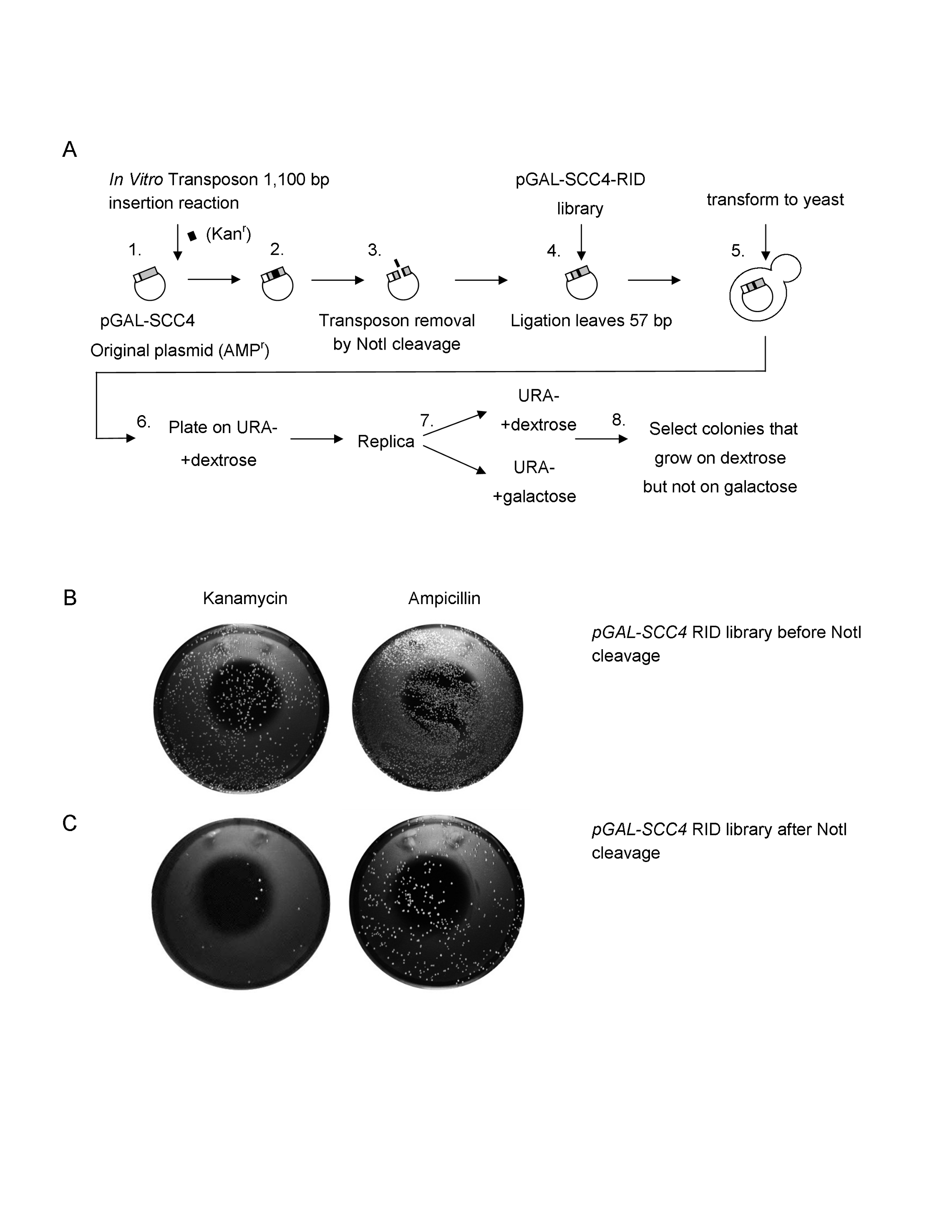

Supplement: Supplemental Material [file supp_g3.116.031674_FigureS1.tif]

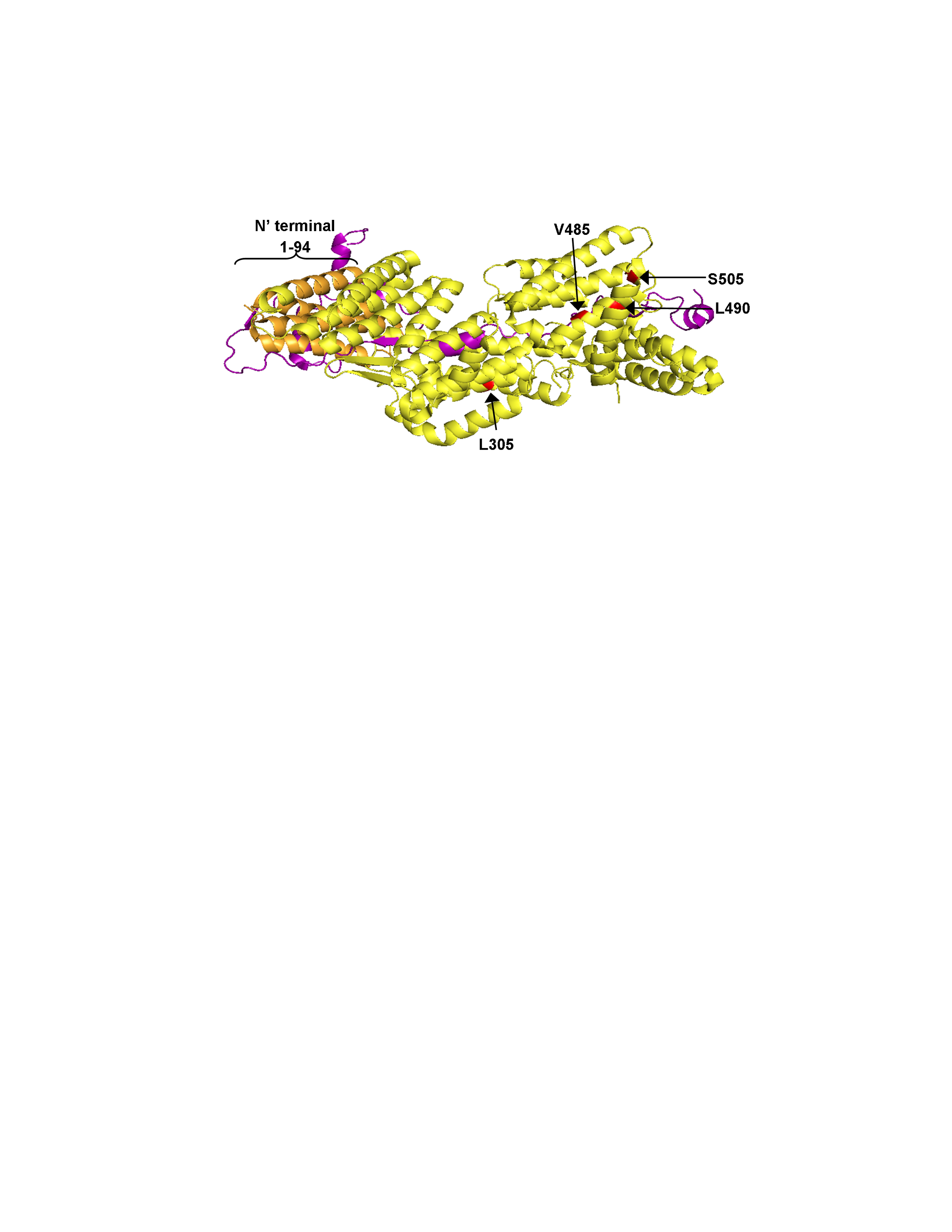

Supplement: Supplemental Material [file supp_g3.116.031674_FigureS2.tif]

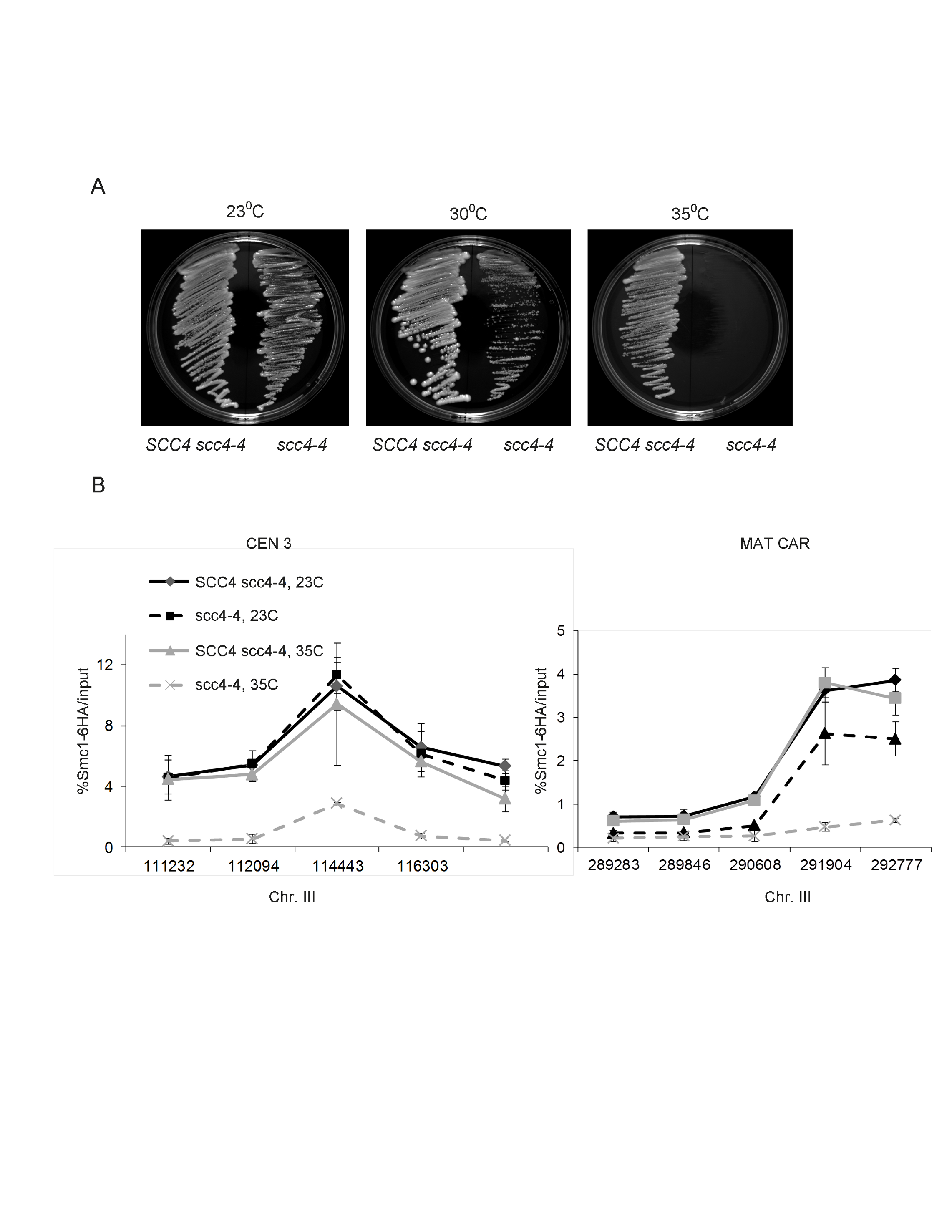

Supplement: Supplemental Material [file supp_g3.116.031674_FigureS3.tif]

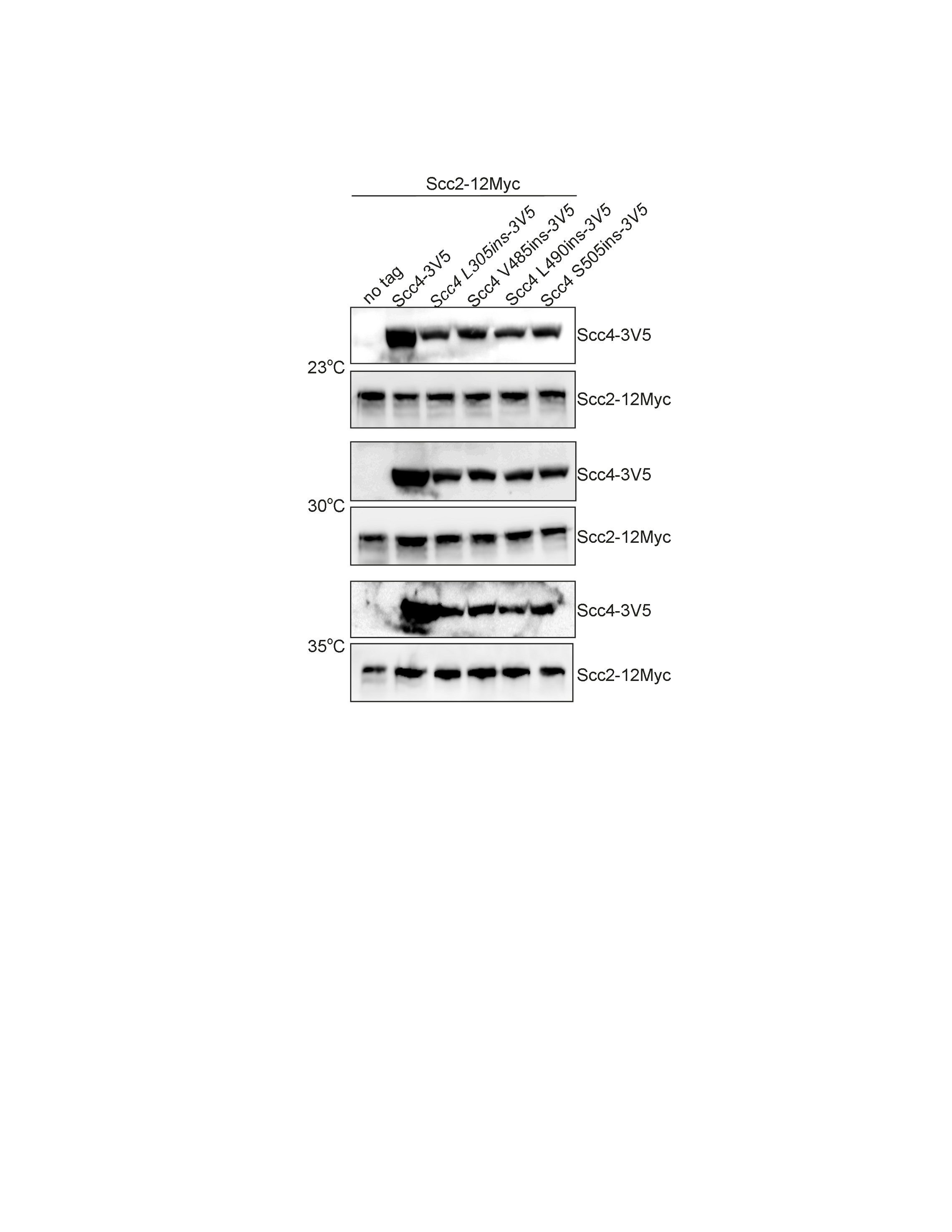

Supplement: Supplemental Material [file supp_g3.116.031674_FigureS4.tif]

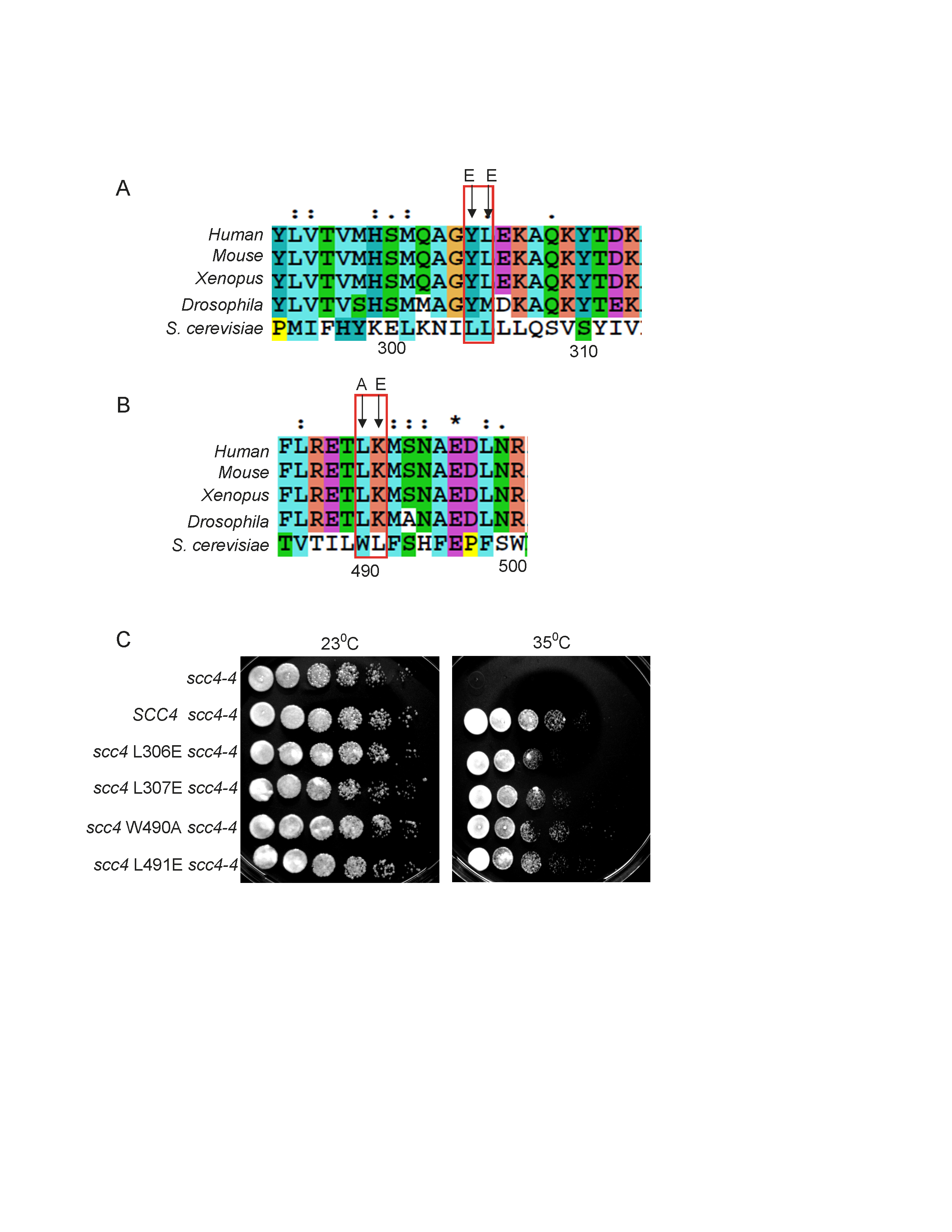

Supplement: Supplemental Material [file supp_g3.116.031674_FigureS5.tif]
